# DUX4 promotes transcription of FRG2 by directly activating its promoter in facioscapulohumeral muscular dystrophy

**DOI:** 10.1186/2044-5040-4-19

**Published:** 2014-10-24

**Authors:** Peter E Thijssen, Judit Balog, Zizhen Yao, Tan Phát Pham, Rabi Tawil, Stephen J Tapscott, Silvère M Van der Maarel

**Affiliations:** 1 Department of Human Genetics, Leiden University Medical Center, Albinusdreef 2, Leiden 2333 ZA, The Netherlands; 2 Division of Human Biology, Fred Hutchinson Cancer Research Center, 1100 Fairview Avenue North, Seattle WA 98109, USA; 3 Neuromuscular Disease Unit, Department of Neurology, University of Rochester Medical Center, Rochester, NY, USA

**Keywords:** Facioscapulohumeral muscular dystrophy (FSHD), *FRG2*, D4Z4, DUX4, Promoter, Transcription factor

## Abstract

**Background:**

The most common form of facioscapulohumeral muscular dystrophy (FSHD) is caused by a genetic contraction of the polymorphic D4Z4 macrosatellite repeat array in the subtelomeric region of chromosome 4q. In some studies, genes centromeric to the D4Z4 repeat array have been reported to be over-expressed in FSHD, including *FRG1* and *FRG2*, presumably due to decreased long-distance repression by the shorter array through a mechanism similar to position-effect variegation. Differential regulation of *FRG1* in FSHD has never been unequivocally proven, however, *FRG2* has been reproducibly shown to be induced in primary FSHD-derived muscle cells when differentiated *in vitro*. The molecular function of FRG2 and a possible contribution to FSHD pathology remain unclear. Recent evidence has identified the mis-expression of *DUX4*, located within the D4Z4 repeat unit, in skeletal muscle as the cause of FSHD. DUX4 is a double homeobox transcription factor that has been shown to be toxic when expressed in muscle cells.

**Methods:**

We used a combination of expression analysis by qRT/PCR and RNA sequencing to determine the transcriptional activation of *FRG2* and *DUX4.* We examined this in both differentiating control and FSHD derived muscle cell cultures or DUX4 transduced control cell lines. Next, we used ChIP-seq analysis and luciferase reporter assays to determine the potential DUX4 transactivation effect on the *FRG2* promoter.

**Results:**

We show that DUX4 directly activates the expression of *FRG2*. Increased expression of FRG2 was observed following expression of DUX4 in myoblasts and fibroblasts derived from control individuals. Moreover, we identified DUX4 binding sites at the *FRG2* promoter by chromatin immunoprecipitation followed by deep sequencing and confirmed the direct regulation of DUX4 on the *FRG2* promoter by luciferase reporter assays. Activation of luciferase was dependent on both DUX4 expression and the presence of the DUX4 DNA binding motifs in the *FRG2* promoter.

**Conclusion:**

We show that the FSHD-specific upregulation of *FRG2* is a direct consequence of the activity of DUX4 protein rather than representing a regional de-repression secondary to fewer D4Z4 repeats.

## Background

Facioscapulohumeral muscular dystrophy (FSHD; OMIM 158900/158901) is one of the most common myopathies with a prevalence of 1 in 8,000 according to a recent report [1]. Individuals with FSHD typically suffer from progressive weakening and wasting of the facial and upper extremity muscles with considerable inter- and intra-familial variability in disease onset and progression [2]. The pathogenic mechanism of FSHD has been linked to the polymorphic D4Z4 macrosatellite repeat array, located on chromosome 4q35, of which each unit contains a copy of a retrogene encoding for the germline transcription factor double homeobox protein 4 (DUX4) [3-5]. DUX4 has been shown to regulate a set of germline, early development, and innate immune response related genes and leads to increased levels of apoptosis when expressed in muscle cells [6-8]. Over recent years, a combination of detailed genetic and functional analyses in FSHD families and muscle biopsies has established that sporadic expression of the *DUX4* retrogene in skeletal muscle is a feature shared by all individuals suffering from FSHD [9,10].

Genetically, at least two forms of FSHD can be recognized. The majority of affected individuals (FSHD1, >95%) are characterized by a contraction of the D4Z4 repeat array. In healthy individuals, the D4Z4 macrosatellite repeat array consists of 11 to 100 copies, whereas individuals with FSHD1 have at least one contracted allele of 1 to 10 repeat units [4,5,11]. A second group of FSHD individuals (FSHD2, <5%) do not show a contraction of the D4Z4 repeat array, however most often have mutations in the chromatin modifier structural maintenance of chromosomes hinge domain containing protein 1 (*SMCHD1*) on chromosome 18p [12]. Both groups share two important (epi-)genetic features: they carry an allele permissive for stable *DUX4* transcription because of the presence of a polymorphic *DUX4* polyadenylation signal (PAS) and they display epigenetic derepression of the D4Z4 repeat array in somatic tissue [9,13]. More specifically, in muscle biopsies and muscle cell cultures DUX4 expression has been correlated with decreased levels of CpG methylation and repressive histone modifications together with increased levels of transcriptional permissive chromatin markers at D4Z4 [10,14-17]. The epigenetic changes at D4Z4 can be either attributed to repeat array contraction (FSHD1) or loss of SMCHD1 activity at D4Z4 (FSHD2) [2].

**Figure 1:**
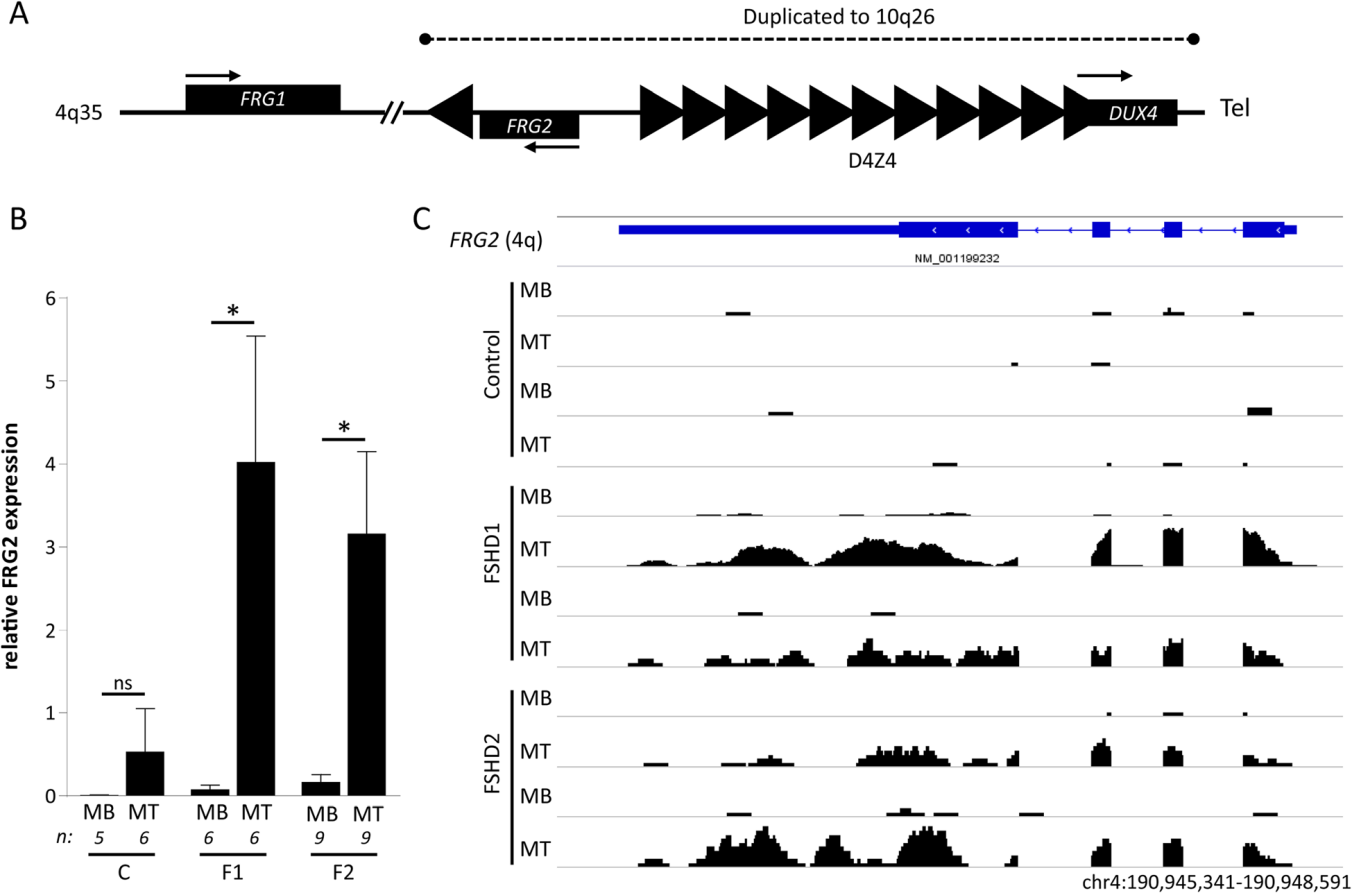
**FRG2 activation in FSHD derived differentiating myoblasts. (A)** Schematic representation of the FSHD locus on chromosome 4q35. Rectangles indicate the different genes, arrows their transcriptional direction. Triangles represent D4Z4 repeat units and the single inverted repeat unit upstream of *FRG2*. Each unit contains the full *DUX4* ORF, only the last repeat unit produces a stable transcript in FSHD patients. The dashed line indicates the duplicated region present on chromosome 10q26. **(B)** qRT-PCR analysis of mean FRG2 expression levels in control (C), FSHD1 (F1) and FSHD2 (F2) derived proliferating myoblasts (MB) and differentiating myotubes (MT) shows the significant activation of *FRG2* during differentiation only in F1 and F2 derived cells. Relative expression was determined using *GAPDH* and *GUSB* as reference genes. Sample numbers are indicated and error bars represent the standard error of the mean. Asterisks indicate significant differences based on a one-way ANOVA (*P* = 0.0014), followed by pairwise comparison using Bonferroni correction, NS = non-significant. **(C)** Genomic snapshot (location indicated at the bottom) of RNA sequencing data of two control, two FSHD1 and two FSHD2 derived proliferating myoblasts (MB) and differentiating myotubes (MT) confirms the full length expression of FRG2 in differentiating myotubes originating from FSHD individuals.

The time interval between the genetic association of FSHD to the D4Z4 macrosatellite repeat array and the identification of sporadic DUX4 activation as a unifying disease mechanism encompasses almost 20 years of research into different candidate genes for FSHD [4,9]. In the absence of a conclusive disease mechanism, genes proximal to the D4Z4 repeat have been investigated as possible FSHD disease genes, postulating that their regulation is affected in FSHD through a position effect emanating from the D4Z4 repeat array [16-22]. Among those candidate genes were FSHD Region Gene 1 (*FRG1*) and FSHD Region Gene 2 (*FRG2*) (Figure 1A). *FRG1* is located on chromosome 4 at 120 kb proximal to the D4Z4 repeat and encodes a protein involved in actin bundle organization and mRNA biogenesis and transport [23-26]. Its overexpression leads to a dystrophic phenotype in different animal models, probably by affecting actin bundling and splicing of transcripts encoding muscle effector proteins [27-30]. However, most studies have failed to demonstrate FRG1 upregulation in FSHD muscle [17,19,20,31-40]. *FRG2* is a gene at 37 kb distance from the repeat encoding a nuclear protein of unknown function [41]. The distal end of chromosome 4 that contains the D4Z4 macrosatellite repeat array has been duplicated to chromosome 10 [42,43]. Consequently, due to its close proximity to the D4Z4 repeat array, *FRG2* is located on both chromosomes 4 and 10 (Figure 1A). Additionally, a complete copy of *FRG2* has been identified on the short arm of chromosome 3. We and others have previously reported on FSHD-specific transcriptional upregulation of *FRG2* from both the 4q and 10q copies upon *in vitro* myogenesis, however its overexpression did not lead to a dystrophic phenotype in a transgenic mouse model [17,27,31,41].

Until the discovery that mis-expression of DUX4 is shared by all FSHD individuals, these observations led to a disease model in which the contraction of the D4Z4 repeat array would create a position effect on proximal genes, thereby leading to their transcriptional activation. Such a mechanism cannot explain the activation of the 10q copy of *FRG2*, as this would require a trans-effect of the contracted repeat array. Although the D4Z4 copies on 4q and 10q have been shown to interact in interphase nuclei [44], this seems unlikely as 3D FISH approaches have revealed that the 4q D4Z4 repeat localizes to the nuclear periphery, whereas the 10q subtelomere does not [45]. More recently, it was shown that the expression of *FRG2* in FSHD cells was influenced by telomere length through telomere position effects [46], leading to the conclusion that *DUX4* and *FRG2* were independently regulated by telomere-length. In the current study we provide evidence that the activation of *FRG2* is a direct consequence of DUX4 protein activity, providing an experimentally supported cause for its specific expression in FSHD muscle and reconfirming DUX4 as the FSHD disease gene.

## Methods

### Cell culture

Human primary myoblast cell lines were obtained from the University of Rochester biorepository (http://www.urmc.rochester.edu/fields-center/) and were expanded and maintained in DMEM/F-10 (#31550 Gibco/Life Technologies, Bleiswijk, The Netherlands) supplemented with 20% heat inactivated fetal bovine serum (FCS #10270 Gibco), 1% penicillin/streptomycin (#15140 Gibco), 10 ng/mL rhFGF (#G5071 Promega, Leiden, The Netherlands) and 1 μM dexamethasone (#D2915 Sigma-Aldrich, Zwijndrecht, The Netherlands). Differentiation into myotubes was started at 80% confluency by serum starvation in DMEM/F-12 Glutamax (#31331, Gibco) supplemented with 2% KnockOut serum replacement formulation (#10828 Gibco) for 36 h. All samples and their characteristics used for our study are listed in Additional file 1: Table S1. Rhabdomyosarcoma TE-671 were maintained in DMEM (#31966) supplemented with 10% FCS and 1% P/S (all Gibco).

### RNA isolation, cDNA synthesis, and qRT-PCR

Cells were harvested using Qiazol lysis reagent (#79306 Qiagen N.V., Venlo, The Netherlands) and RNA was subsequently isolated using the miRNeasy Mini Kit (#217004 Qiagen) including an on column DNase treatment according to the manufacturer’s instructions. A total of 2 μg RNA was used to synthesize poly-dT primed cDNA using the RevertAid H Minus First strand cDNA Synthesis Kit (#K1632 Thermo Fischer Scientific Inc., Waltham, MA, USA). Relative FRG2 expression was quantified on the CFX96 system (Bio-Rad, Veenendaal, The Netherlands) using SYBR green master mix (Bio-Rad) with the following primers: FRG2_Fw: GGGAAAACTGCAGGAAAA, FRG2_Rv: CTGGACAGTTCCCTGCTGTGT. For relative quantification *GUSB* and *GAPDH* were used as reference genes and amplified with the following primers: GAPDH_Fw: GAGTCAACGGATTTGGTCGT, GAPDH_Rv: TTGATTTTGGAGGGATCTCG, GUSB_Fw: CCGAGTGAAGATCCCCTTTTTA, GUSB_Rv: CTCATTTGGAATTTTGCCGATT. All PCR reactions were carried out in duplicate and the data were analyzed using the Bio-Rad CFX manager version 3.0 (Bio-Rad).

### Luciferase reporter assays

Genomic fragments containing the *FRG2* promoter were amplified by regular PCR (primers pFRG2 _Fw: AGGCCTTACCTTGCCTTTGT; pFRG2_Rv: TCTTGCTGGTGGATGTTGAG) using cosmids 23D11 (chromosome 10) and cY34 (chromosome 4) [47,48]. The obtained PCR fragments containing the promoter sites were digested with BglII and BclI and subcloned into the BglII digested pGL3 basic vector. Genomic locations of the cloned promotersites (UCSC hg19): chr4:190,948,283-190,949,163 and chr10:135,440,170-135,441,050. DUX4 binding sites were deleted by ligating PCR products obtained with internal primers (Fw_internal: ATCTGAGGGCCCTGATTCCTGAGGTAGC, Rv_internal: ATCTGAGGGCCCCATTTTTAAGGTAGGAAGG) combined with RV3 and GL2 primers annealing in the pGL3 backbone. Single binding sites were destroyed using site directed mutagenesis by PCR amplifying overlapping fragments with the following primers: Site 1Fw: CCTCAGGAATCAGGGGCTACATAGGGTAGCACTGACTCAACCT, Site 1Rv: AGGTTGAGTCAGTGCTACCCTATGTAGCCCCTGATTCCTGAGG, Site 2Fw: GGCTAATTAGGTTAGCACTGACTCACCCTATGCAATTCAATTTTATTGCATTTGATC, Site 2Rv: GATCAAATGCAATAAAATTGAATTGCATAGGGTGAGTCAGTGCTAACCTAATTAGCC, Site 3Fw: ACCTAATCAATTCAATTTTATTGCATTTGCACTAAGTATCTTCCCCATTTTTAAGGTAGGAAGG, Site 3Rv: CCTTCCTACCTTAAAAATGGGGAAGATACTTAGTGCAAATGCAATAAAATTGAATTGATTAGGT together with RV3 and GL2 primers. Insert sequences and correct orientation in the pGL3 vector were confirmed by Sanger sequencing. Sixty thousand TE671 cells were seeded in standard 24 well tissue culture plates and co-transfected with 200 ng pCS2/pCS2-DUX4 and 200 ng of the indicated pGL3 constructs, using lipofectamine 2000 according to manufacturer’s instructions. Twenty-four hours after transfection cell lysates were harvested and luciferase activity was measured using the Promega luciferase assay kit, according to manufacturer’s instructions. Co-transfection with Renilla luciferase constructs for data normalization was omitted as we previously observed regulation of this construct by DUX4 [49]. Transfections were carried out in triplicate, error bars indicate the SEM of three independent experiments.

### RNA sequencing and ChIP sequencing

RNA sequencing and ChIP sequencing data were obtained and analyzed as described before [6,36]. All datasets have previously been made publicly available in the Gene Expression Omnibus (accession numbers GSE56787, GSE33838). FRG2 expression in response to DUX4 overexpression is displayed for MB135, a control derived primary myoblast, and a control derived fibroblast. The genomic snapshots of the different datasets were generated using the IGV genome viewer version 2.3.32 [50,51].

## Results

### FRG2 expression is activated in differentiating FSHD derived muscle cells

**Figure 2:**
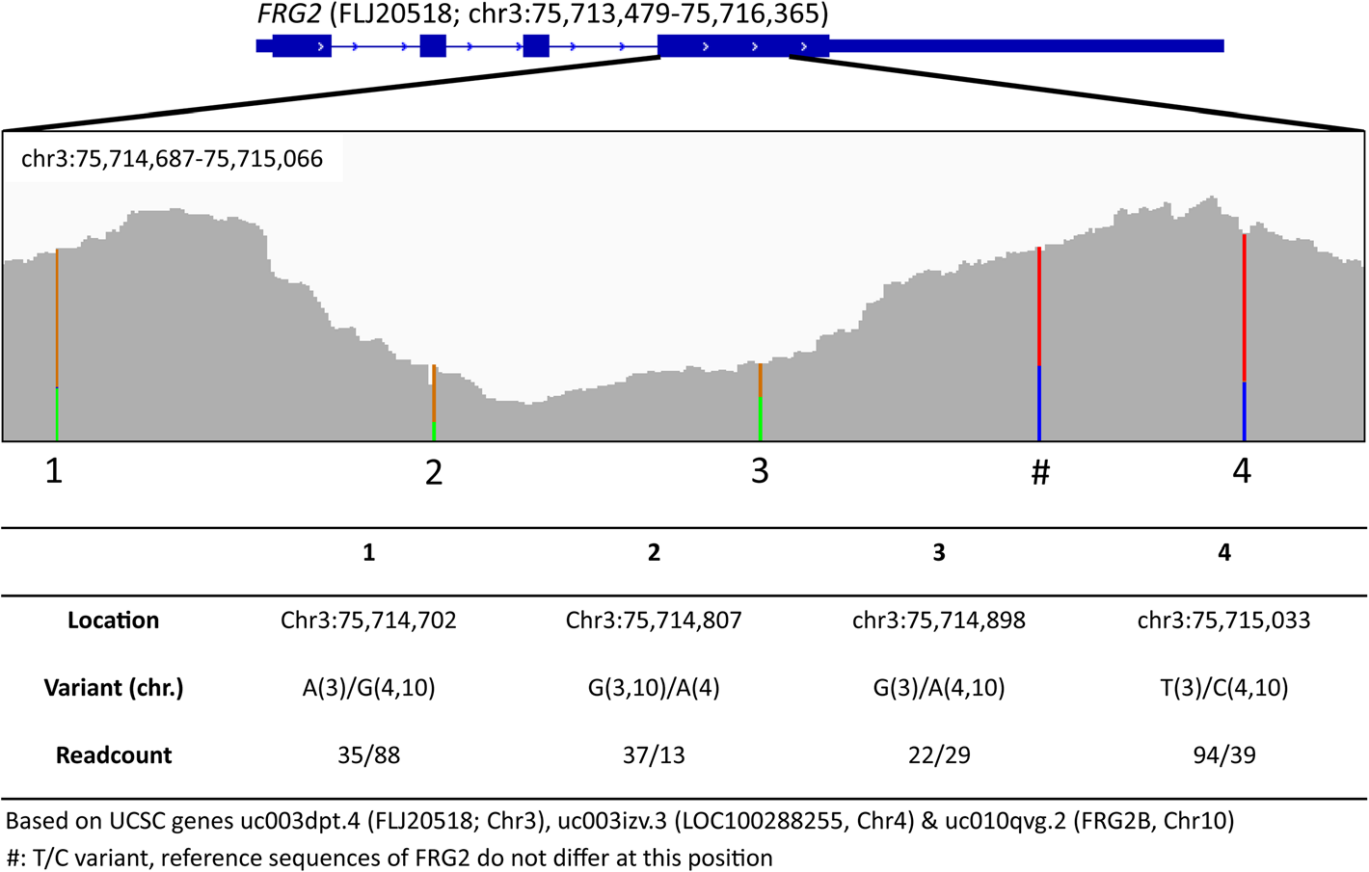
**Sequence analysis of RNA sequencing reads reveals activation of FRG2 from all copies.** Graphical representation of RNA-seq reads mapping to the *FRG2C* locus at chromosome 3p. Single nucleotide polymorphisms can be identified and are indicated by colored vertical lines (A = green, C = blue, G = orange, T = red). Different reads could thereby be assigned to the three different genomic copies of *FRG2*. Sequence analysis was based on the reference sequences obtained from the UCSC genome browser (build 19).

We cultured a set of primary muscle cells derived from six controls, six FSHD1, and nine FSHD2 individuals and harvested RNA to analyze FRG2 transcript levels by quantitative realtime-PCR (qRT-PCR). We confirmed the significant FSHD-specific activation of *FRG2* in differentiating myotubes (Figure 1B). In control samples, we observed a minor increase in *FRG2* expression that was not statistically significant (Figure 1B). Analysis of previously published RNA-seq data from two additional controls and a subset of FSHD samples confirmed *FRG2* activation (Figure 1C) and single base pair variations, known to differ between the three copies of *FRG2*[41], indicated transcripts were induced from *FRG2* genes at all three genomic locations (Figure 2). Therefore, we conclude that increased transcription of *FRG2* is not restricted to the copy on the 4q disease allele in FSHD1 and likely not caused by a *cis* effect of D4Z4 chromatin relaxation. The increase of FRG2 transcript levels coincided with activation of DUX4 transcription upon *in vitro* myogenesis in FSHD derived samples (Additional file 2: Figure S1), as was reported before by us and others [36,52,53]. Altogether this confirms previously published data and highlights the robust transcriptional activation of all annotated copies of *FRG2* in differentiating FSHD myotubes.

### FRG2 activation is a direct consequence of DUX4 protein activity

**Figure 3:**
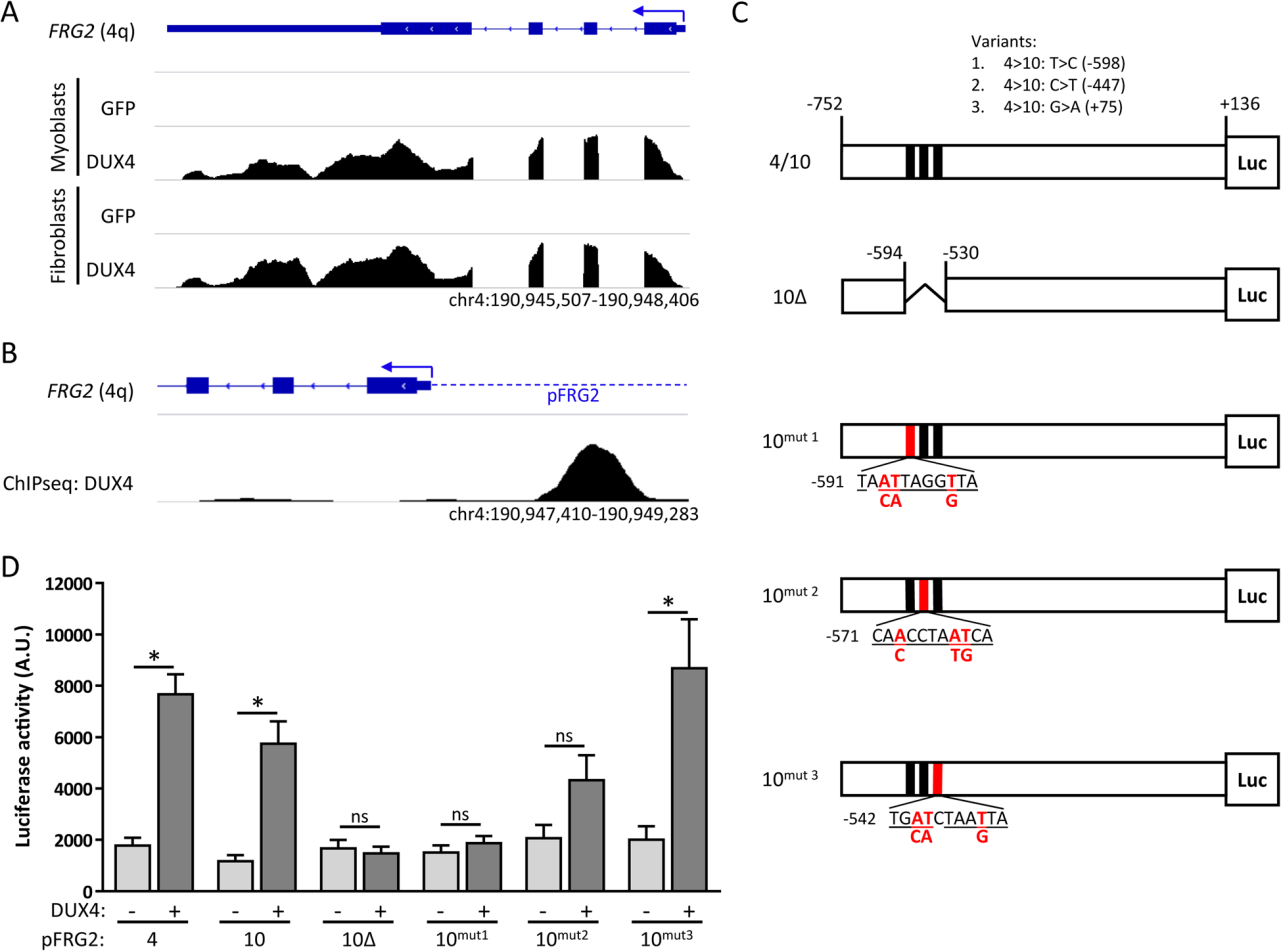
**FRG2 is activated as a consequence of DUX4 protein activity at its promoter. (A)** Genomic snapshot (location indicated at the bottom) of mapped RNA-seq reads at the chromosome 4q *FRG2* locus. Overexpression of DUX4 results in the activation of *FRG2* in myoblasts and fibroblasts, GFP overexpression was used as a control. **(B)** Graphical representation of DUX4 binding at the 4q FRG2 promoter (genomic snapshot location indicated at the bottom) as revealed by ChIP-seq analysis (data obtained in myoblasts). **(C)** Genomic fragments obtained from chromosomes 4 and 10 were cloned upstream of the luciferase gene. Polymorphisms distinguishing both copies are indicated (Variants 1-3) and the three identified DUX4 binding sites are displayed, with nucleotides matching the previously identified core DUX4 binding sequence underlined. All numbers show the relative distance to the TSS of *FRG2*, in the 10^mut1-3^ constructs the number indicates the first displayed nucleotide. The 10Δ construct lacks all three DUX4 binding sites, whereas in 10^mut1-3^ the red nucleotides were mutated to the nucleotides indicated below them, thereby destroying the individual DUX4 binding sites. **(D)** DUX4 activates the *FRG2* promoter in a luciferase reporter assay. Both the 4q and 10q copy of the *FRG2* promoter are activated by DUX4. The 10q copy lacking the DUX4 binding sites (10Δ) was not activated by DUX4. Destruction of the three individual binding sites revealed that sites 1 and 2 are mediating the DUX4 dependent activation of *FRG2*. Counts per second (CPS) are a direct measure of luciferase activity, error bars indicate the SEM of three independent experiments. Asterisks indicate significant differences based on a one-way ANOVA (*P* <0.0001), followed by pairwise comparison using Bonferroni correction. NS = non-significant.

As the activation of *FRG2* in FSHD-derived myotubes follows the pattern of previously identified DUX4 target genes, we wondered if *FRG2* is regulated by DUX4 directly. Indeed, we previously showed that FRG2 transcription was induced at least two-fold by expression array analysis in DUX4 over-expressing control myoblasts [6]. This robust increase in FRG2 transcription was confirmed by RNA-seq in both myoblasts and fibroblasts (Figure 3A) that were transduced with DUX4 expressing lentiviruses, ruling out a muscle specific effect of DUX4 on *FRG2* expression. Direct targets of DUX4 were previously identified by overlaying expression data with chromatin immuno-precipitation (ChIP) sequencing data [6]. Following the same approach, we observed DUX4 binding at the promoter of *FRG2* (Figure 3B). Sequence analysis of the 4q and 10q copies of the *FRG2* promoter revealed that both chromosomes harbor three consensus binding sites for DUX4, which are not affected by minor sequence differences between the two loci (Figure 3C). To test the functional significance of these DUX4 binding sites, we designed luciferase reporter constructs harboring the *FRG2* promoters of chromosomes 4 and 10. Co-transfection of these constructs with pCS2-DUX4 or the empty pCS2 backbone in TE-671 rhabdomyosarcoma cells confirmed that the activation of *FRG2* is mediated by DUX4 protein expression (Figure 3D). To confirm that the activation of the luciferase reporter gene was indeed mediated by DUX4 binding, we generated a reporter construct with a micro-deletion of all three DUX4 binding sites in the *FRG2* promoter derived from chromosome 10 (10Δ, Figure 3C). Upon co-transfection with the PCS2-DUX4 expression vector, the luciferase activation was completely ablated (Figure 3D). We identified three consecutive DUX4 binding sites in the *FRG2* promoter, of which sites one and three contain a single base pair variation in the core sequence identified previously [6]. To dissect which sites were responsible for DUX4 dependent *FRG2* activation, we designed three additional constructs, in which one of the three sites was destroyed by site directed mutagenesis (Figure 3C). Upon transfection of these constructs we observed that the activation of *FRG2* by DUX4 is mediated primarily through the two binding sites furthest from the *FRG2* transcriptional start site (TSS), as luciferase activity in response to DUX4 expression was no longer significantly induced. While destruction of the first binding site resulted in a complete absence of luciferase activation, the effect of DUX4 is still moderate, but non-significant, if site two is mutated (Figure 3D). Destroying the third binding site did not affect the DUX4 mediated activation of the *FRG2* promoter (Figure 3D). Altogether, our data show that DUX4 binding at the FRG2 promoter is underlying the transcriptional activation of *FRG2* in FSHD derived myogenic cultures.

## Discussion

Ever since the D4Z4 macrosatellite repeat array was genetically associated with FSHD, great effort has been put into identifying the underlying disease mechanism. The initial lack of evidence for active transcription of *DUX4*, encoded in each D4Z4 repeat unit, shifted the focus to genes immediately centromeric to the array, like *FRG1* and *FRG2*. Although FRG1 overexpression in mice leads to a dystrophic phenotype, its deregulation in muscles of FSHD patients has not been consistently demonstrated [19,27,28,31-34,36-40]. In contrast, upregulation of *FRG2* was consistently reported in FSHD-derived differentiating muscle cells; however the mechanism of FSHD-specific upregulation of FRG2 had not been conclusively established [17,31,41].

Activation of *FRG2* in FSHD cells was previously attributed to de-repression through a position effect mechanism secondary to the contraction of the D4Z4 repeat array [17,41]. Moreover, it was shown that KLF15 regulates both *FRG2* expression and the activity of a putative enhancer in within D4Z4, thereby possibly facilitating a *cis* effect of the D4Z4 repeat array on the proximal *FRG2* locus [54]. This model was challenged by showing that CpG methylation levels at a single CpG site centromeric to D4Z4 are unaffected in FSHD [13], indicating that DNA methylation was not broadly altered in the region centromeric to the D4Z4 repeat array in FSHD cells. The recent establishment of a unifying disease mechanism for FSHD, that primarily centers around DUX4, further challenges a position effect model that involves the deregulation of genes proximal to D4Z4 as a consequence of D4Z4 repeat array contraction. In line with this notion, we now show that *FRG2* is a direct target gene of DUX4, providing a direct explanation for its upregulation in FSHD muscle.

As sporadic *DUX4* activation is induced during *in vitro* muscle cell differentiation, the expression profile of *FRG2* fits that of previously reported DUX4 target genes. It is interesting to note that we observed increased, though non significant, FRG2 expression in control derived differentiating muscle cells, which might indicate a minimal activation of the locus during *in vitro* myogenesis in control cells. DUX4 expression has been reported to occur in control derived myogenic cultures, albeit at much lower frequencies, and thus FRG2 may be activated as a consequence of that [55]. Alternatively, FRG2 expression may be sporadically induced through other mechanisms, exemplified by the reported regulation by KLF15 [54].

DUX4 acts a potent transcriptional activator and induces expression of germline and early development genes through binding a specific homeobox sequence [6]. ChIP-seq analysis indeed identified DUX4 binding at the promoter of *FRG2*, which contains three consecutive DUX4 binding sites. Our experiments showed that the two sites furthest from the TSS of *FRG2* are mediating the activation of *FRG2* upon the expression of DUX4, confirming our earlier work showing that the probability of transcriptional activation by DUX4 increases with the number of consecutive DUX4 binding sites [56]. The FRG2 promoter sites at chromosomes 3, 4, and 10 are highly conserved, with identical sequences for the DUX4 sites on 4 and 10 and a limited number of single nucleotide differences between these and chromosome three that are predicted to preserve DUX4 binding, explaining the activation of all copies by DUX4 [41]. In contrast to the complicated proposed mechanism of *cis* and *trans* effects of D4Z4 contraction at 4q [17,41], the protein activity of DUX4 offers a simple and experimentally supported explanation for the activation of *FRG2.*

It was previously shown that FRG2 and DUX4 are regulated, at least in part, by telomere position effects [46]. *Trans*-activation of the *FRG2* promoter was ruled out by transfecting promoter reporter constructs in immortalized FSHD derived myoblasts. However, the sporadic nature of *DUX4* expression in these cells would seriously decrease the signal-to-noise ratio in this assay and a direct effect of DUX4 on FRG2 expression would likely have been missed. Although we cannot rule out a direct telomere position effect on *FRG2* in this study, we suggest that the observed increase of FRG2 can be attributed to the increased DUX4 levels rather than to telomere position effects on FRG2. DUX4 itself may indeed be partially under control of telomere length and as such its target genes would follow a similar regulation.

As of yet, the functional consequence of *FRG2* activation in FSHD remains elusive. FRG2 localizes to the nucleus [41], but its function has never been demonstrated and a possible role in FSHD disease progression is therefore unclear. The identification of FSHD individuals with proximally extended deletions, in which not only a large part of the D4Z4 repeat array, but also proximal sequences (including *FRG2*) were deleted, again suggests that a cis-acting effect on *FRG2* is not necessary for FSHD pathology [57,58]. However, since DUX4 activates other genomic copies of *FRG2*, it remains possible that this protein contributes to some aspect of the disease.

## Conclusion

In this study we have firmly established that the long known activation of *FRG2* in FSHD derived differentiating muscle cells is a direct consequence of DUX4 activity at its promoter. This provides further evidence for DUX4 as the central player in the FSHD disease mechanism and demonstrates that the higher expression of *FRG2* in FSHD does not result from regional de-repression secondary to fewer D4Z4 repeats.

## Abbreviations

FSHD: Facioscapulohumeral muscular dystrophy; FRG1: FSHD Region Gene 1; FRG2: FSHD Region Gene 2; DUX4: Double homeobox 4; SMCHD1: Structural maintenance of chromosomes flexible hinge domain containing 1; PAS: Polyadenylation signal; FISH: Fluorescence in situ hybridization; FBS: Fetal bovine serum; rhFGF: Recombinant human fibroblast growth factor; SEM: Standard error of the mean; ChIP-seq: Chromatin immunoprecipitation followed by deep sequencing; qRT-PCR: Quantative realtime polymerase chain reaction; RNA-seq: RNA sequencing; KLF15: Krüppel-like factor 15; ORF: Open reading frame; MB/MT: Myoblasts/myotubes; TSS: Transcriptional start site; ANOVA: Analysis of variance; CPS: Counts per second.

## Competing interests

The authors declare that they have no competing interests.

## Authors’ contributions

PT designed, performed, and analyzed experiments, analyzed genome-wide data, and drafted and finalized the manuscript; JB designed, performed, and analyzed experiments, and revised the manuscript; ZY designed, performed, and analyzed genome-wide experiments/data, and revised the manuscript; TPP designed, performed, and analyzed experiments; RT provided key biological material and revised the manuscript; SJT designed and analyzed experiments, and drafted and revised the manuscript; SVDM designed and analyzed experiments, and drafted and revised the manuscript. All authors read and approved the final manuscript.

## Supplementary Material

Additional file 1: Table S1 Characteristics of samples used for qRT-PCR and RNA-seq analysis. Length in kb and haplotype of both 4q D4Z4 alleles are indicated. Click here for file

Additional file 2: Figure S1 RNA-sequencing reads mapping to D4Z4 showed DUX4 activation in differentiating FSHD derived muscle cells. Graphical representation of RNA-seq reads mapping to the 4q D4Z4 repeat, indicative for DUX4 expression, showed FSHD specific activation of DUX4 upon differentiation. Sequenced transcripts were mapped to DUX4 ORFs, encoded within each D4Z4 repeat, which are annotated with gene symbols DUX2, DUX4, DUX4L4, and DUX4L5. MB = myoblasts, MT = myotubes; the genomic location of the snapshot is indicated at the bottom. Click here for file
