# Supplementary material for: DUX4 promotes transcription of FRG2 by directly activating its promoter in facioscapulohumeral muscular dystrophy
Source: Skelet Muscle. 2014 Oct 24;4:19. doi: 10.1186/2044-5040-4-19 (PMC4364343; doi:10.1186/2044-5040-4-19)
Supplement: Additional file 1: Table S1 — Characteristics of samples used for qRT-PCR and RNA-seq analysis. Length in kb and haplotype of both 4q D4Z4 alleles are indicated. [file 2044-5040-4-19-S1.pdf]

Group	#	Gender	Age at biopsy	D4Z4 alleles (chromosome 4):	qRT-PCR	RNA-seq
Control	1	male	37	94kb 168B, 128kb 161A	x	
Control	2	female	44	28kb 163B, 65kb 161A	x	
Control	3	male	58	47kb 161A, 74kb 168B	x	
Control	4	male	65	85kb 168B, 166kb 163B	x	
Control	5	male	29	47kb 161A, 72kb 168B	x	
Control	6	male	62	47kb 161A, 72kb 168B	x	
Control	7	male	40	65kb A161, 101kb B163		x
Control	8	female	42	65 kb 163B, 250kb 161A		x
FSHD1	1	female	30	23kb 161A, 178kb 168B	x	x
FSHD1	2	female	46	27kb 161A, 87kb 163B	x	
FSHD1	3	female	47	19kb 161A, 47kb 163B	x	
FSHD1	4	female	41	27kb 161A, 90kb 162B	x	
FSHD1	5	female	60	26kb 161A, 82kb 168B	x	
FSHD1	6	male	33	28kb A161, 44kb H168A	x	x
FSHD2	1	male	44	43kb 161A, 132kb 163B	x	
FSHD2	2	male	36	44kb 161A, 63kb 161A	x	
FSHD2	3	male	59	50kb 161A, 58kb 163B	x	
FSHD2	4	female	69	47kb 161A, 76kb 161A	x	x
FSHD2	5	male	42	90kb 161A, 131kb 166A	x	
FSHD2	6	male	26	54kb 161A, 220kb 161A	x	
FSHD2	7	male	34	54kb 161A, 70kb 161A	x	
FSHD2	8	female	56	67kb 161A, 337kb 168B	x	x
FSHD2	9	male	28	65kb 161A, 160kb 161A	x	

Additional table 1: Characteristics of samples used for qRT-PCR and RNA-seq analysis. Length in kb and haplotype of both 4q D4Z4 alleles are indicated.
