# Supplementary figures and images for: DUX4 promotes transcription of FRG2 by directly activating its promoter in facioscapulohumeral muscular dystrophy
Source: Skelet Muscle. 2014 Oct 24;4:19. doi: 10.1186/2044-5040-4-19 (PMC4364343; doi:10.1186/2044-5040-4-19)

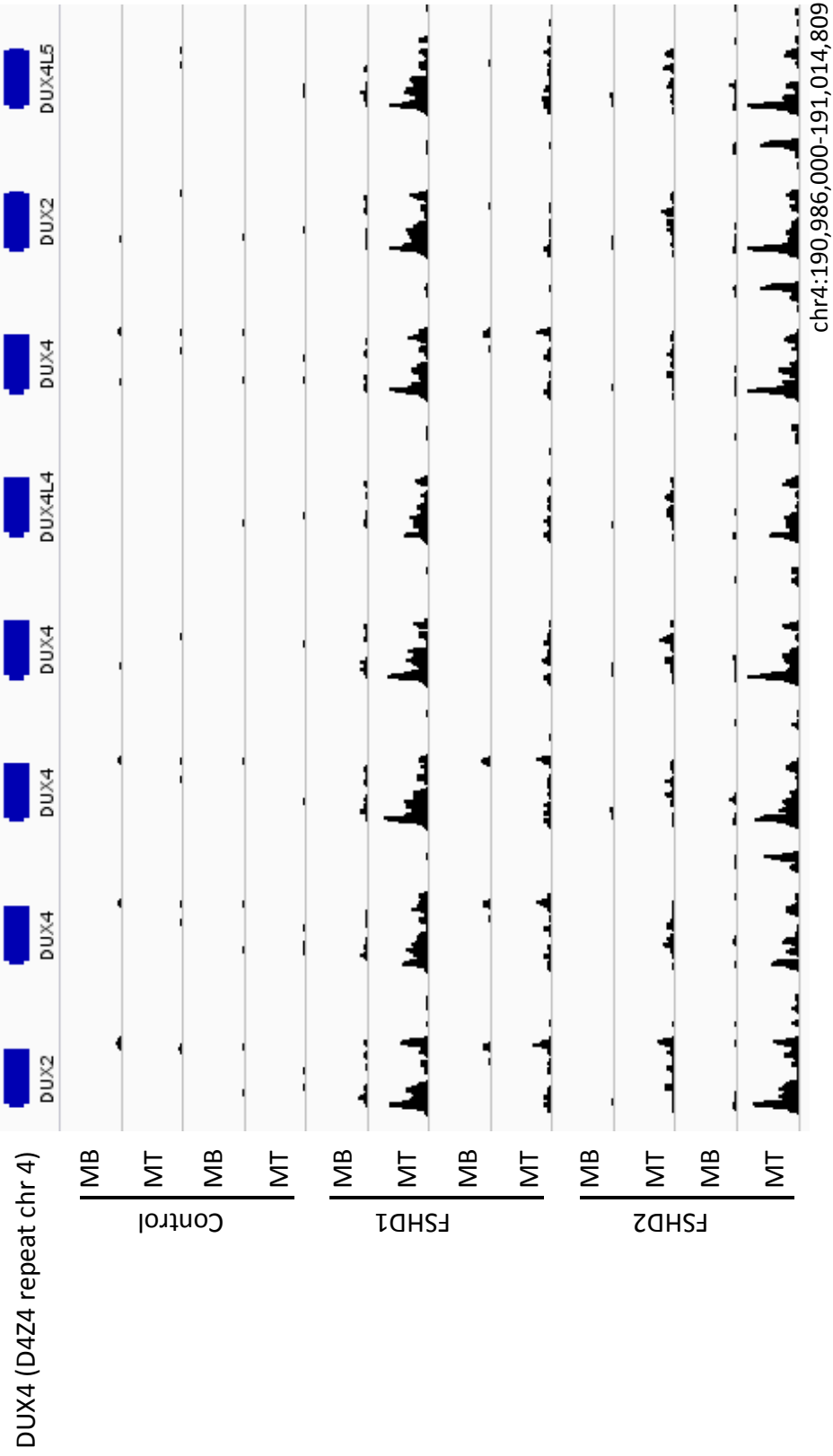

Supplement: Additional file 2: Figure S1 — RNA-sequencing reads mapping to D4Z4 showed DUX4 activation in differentiating FSHD derived muscle cells. Graphical representation of RNA-seq reads mapping to the 4q D4Z4 repeat, indicative for DUX4 expression, showed FSHD specific activation of DUX4 upon differentiation. Sequenced transcripts were mapped to DUX4 ORFs, encoded within each D4Z4 repeat, which are annotated with gene symbols DUX2, DUX4, DUX4L4, and DUX4L5. MB = myoblasts, MT = myotubes; the genomic location of the snapshot is indicated at the bottom. [file 2044-5040-4-19-S2.pdf]
